# Biofilm formation by the global outbreak strain of *Mycobacterium chimaera* results in significantly reduced efficacy of standard disinfectants

**DOI:** 10.1186/s12866-025-04439-w

**Published:** 2025-11-13

**Authors:** Anna Maria Oschmann, Katharina Konrat, Christoph Schaudinn, Genevieve Sohl, Dirk Wagner, Astrid Lewin, Mardjan Arvand

**Affiliations:** 1 Hospital Hygiene, Infection Prevention and Control, Robert Koch Institute, Nordufer 20, 13353 Berlin, Germany; 2 Mycotic and Parasitic Agents and Mycobacteria, Robert Koch Institute, Berlin, Germany; 3 Advanced Light and Electron Microscopy, Robert Koch Institute, Berlin, Germany; 4 Division of Infectious Diseases, Department of Internal Medicine II, Freiburg University Medical Center, Freiburg, Germany; 5 Department of Infectious Diseases, Medical Microbiology and Hygiene, Heidelberg University, Heidelberg, Germany

**Keywords:** Mycobacteria, Nontuberculous, Outbreak, Biofilm, Contamination, Disinfection, Heater-cooler device, Nosocomial, Transmission

## Abstract

**Background:**

In 2013, a global outbreak of *Mycobacterium chimaera* infections due to contaminated heater-cooler units emerged. This ongoing problem has highlighted the question of whether disinfection recommendations for medical devices containing water circuits are adequate for preventing contamination and possible recontamination by nontuberculous mycobacteria. The formation of biofilms in such devices exacerbates the problem. This study aimed to assess the efficacy of disinfectants on biofilms and suspensions of the *M. chimaera* strain ZUERICH-1, and to compare it with two unrelated *M. chimaera* strains obtained from different sources.

**Methods:**

Disinfection efficacy testing for biofilm was performed using a Bead Assay for Biofilms and for bacteria in suspension according to the European Standard EN 14348. Three different disinfectants, glutaraldehyde, sodium hypochlorite and peracetic acid, were assessed. *M. chimaera* ZUERICH-1, two genetically unrelated *M. chimaera* isolates and *M. avium* subsp. *avium* ATCC 15769, which is included in European standards for disinfectant testing on mycobacteria, were analyzed. The biofilms’ structure and composition were analyzed by chemical and molecular techniques and advanced imaging methods.

**Results:**

We found that peracetic acid and glutaraldehyde in standard concentrations were able to effectively inactivate (≥ 4 log_10_ reduction) suspended bacteria of all three strains, but chlorine failed in all cases. Formation of biofilm generally enhanced the tolerance of *M. chimaera* to disinfectants. Peracetic acid in standard concentration could not effectively inactivate biofilms of *M. chimaera* ZUERICH-1, but was effective against biofilms of the other *M. chimaera* strains tested. Similarly, glutaraldehyde in standard concentration could not inactivate biofilm of ZUERICH-1. Biomass analysis showed higher amounts of extracellular matrix of ZUERICH-1 when compared to the other two strains.

**Conclusions:**

The data suggest that current standard disinfection recommendations do not ensure sustained inhibition of *M. chimaera* when embedded in biofilm. Additional measures are needed to prevent nosocomial transmission of *M. chimaera* through contaminated heater-cooler units.

**Supplementary Information:**

The online version contains supplementary material available at 10.1186/s12866-025-04439-w.

## Introduction

The prevalence of infections caused by nontuberculous mycobacteria (NTM) has increased globally for several years [1]. The contamination of medical devices or surgical material by NTM contributes to healthcare associated infections by these pathogens [2–4]. Since 2013, an ongoing global outbreak of systemic NTM infections has been occurring as a result of contaminated heater–cooler units (HCU). The machines were contaminated by *Mycobacterium chimaera* [5, 6], which belongs to the *Mycobacterium avium* complex (MAC) [7]. It thereby shares typical characteristics of mycobacteria, such as a high natural tolerance to antimicrobials (including disinfectants). During cardiopulmonary bypass surgery, HCU are used to keep the patients’ blood at a specific temperature. The open ventilation system of some HCU can lead to the spread of *M. chimaera* from the contaminated water-cooling circuit via bio-aerosols in the operating room, presumedly resulting in contamination of surfaces, surgical sites and/or prosthetic materials [8]. In 2013, first cases of postoperative prosthetic-valve endocarditis (PVE) resulting from disseminated infections with *M. chimaera* were identified [9]. As of 2018, a global total of > 140 patients has been linked to the outbreak with a fatality rate of approximately 50% [10]. Van Ingen et al. [11] could track the global outbreak back to certain types of HCU that were contaminated at the production site. In the follow, recommendations were issued to improve the microbiological quality of the HCU´s operating water and for cleaning and disinfection of the devices. The guidelines for the disinfection of HCU often rely on oxidative agents that can only be used in a limited concentration due to the degradation of the devices’ internal materials. At the beginning of the outbreak, the disinfection recommendations for the outbreak-related heater – cooler units T3 from Sorin suggested a weekly 10 min disinfection of the water tanks and tubes with either sodium hypochlorite (0.02%) or peracetic acid (0.045%) as reactive agents [12]. Over time, the procedures were adjusted to respond to the ongoing outbreak by increasing the concentrations of the disinfectants (0.1% sodium hypochlorite or 0.1% peracetic acid for 10 min). Updated disinfection procedures as well as intensive cleaning and the replacement of contaminated tubes still could not prevent reemerging growth of the bacteria [13]. This raised the question of whether disinfection procedures can successfully prevent the growth or re-proliferation of *M. chimaera* in such machines at all, which was also complicated by formation of biofilms within the HCU water circuits. This has led to further studies which analyzed different methods for disinfection of HCUs [14].

Biofilms are communities of microorganisms that embed themselves in an extracellular matrix (ECM). They are considered for many microbes to be the most naturally occurring homeostatic lifestyle [15]. The ECM can differ greatly in composition and structure amongst species, strains, as well as living conditions. Several functions have been linked to the biofilm matrix, including enabling communication within the bacterial community, generating a self-sustaining source of nutrition, and providing protection [16, 17]. Growth of biofilms has been shown to enhance the tolerance of many bacterial species to antimicrobial substances. Bridier et al. [18] reviewed disinfection procedures for biofilms and found that biofilm assembly generally leads to increased tolerance to disinfectants. Recent studies have also reviewed and analysed biofilm formation of mycobacteria and it’s impact on tolerance to disinfectants [19, 20].

Since strong biofilm formation in contaminated HCU was reported in the context of the outbreak, we hypothesized that biofilms of *M. chimaera* might behave differently towards disinfectants than single bacteria in suspension and that the *M. chimaera* outbreak strain might behave differently in comparison to other *M. chimaera* strains. We therefore aimed to analyze the efficacy of disinfectants commonly used in HCU on both the suspension and biofilm of *M. chimaera*.

## Materials and methods

### Bacterial strains

The *M. chimaera* outbreak strain ZUERICH-1 and two unrelated *M. chimaera* isolates obtained from different sources (water or clinical specimen) were used in this study. The *M. avium* subsp. *avium* strain ATCC 15769, which is included in European standards for disinfection testing on mycobacteria, was used as an internal control in this study. All strains and their sources are shown in Table 1.

**Table 1:** Strains tested for disinfectant efficacy testing. *M. chimaera* strains of different origin were analyzed. The *M. avium* type strain was used as an internal control for disinfectant testing

Strain	Origin	Source
*M. chimaera* ZUERICH-1 (outbreak strain)	Patient infected during heart surgery in presence of HCU	DSMZ 101591, German Collection of Microorganism and Cell Cultures
*M. chimaera* FR-35	Water tank of HCU (unrelated to outbreak)	Division of Infectious Diseases, Department of Internal Medicine II, Medical Center, University of Freiburg, Freiburg, Germany
*M. chimaera* UP-11	Patient respiratory sample (unrelated to outbreak)	Division of Infectious Diseases, Department of Internal Medicine II, Medical Center, University of Freiburg, Freiburg, Germany
*M. avium* subsp. *avium* ATCC 15769	Spleen of a tuberculous hen	DSMZ 44157, German Collection of Microorganism and Cell Cultures

### Cultivation of bacteria and generation and processing of biofilms

Biofilms were cultivated on porous glass beads and processed as described before [20, 21] with some adjustments for use with mycobacteria. The bacterial strains were stored at −80 °C in 10% glycerol (stock). After thawing, a suspension of approx. 1 × 10^5^ CFU/mL was produced in Middlebrook 7H9 broth supplemented with 10% OADC (BD, Heidelberg, Germany). Porous glass beads (8 mm, ROBU® Glasfilter-Geräte, Hattert, Germany) were placed in 24-well plates (one bead per well), to which 1 mL of the bacterial suspension was added. The plates were incubated on an 8 mm orbital shaker at 150 rpm and 37 °C for as long as required based on experimental parameters. All experiments were performed at least three times, with three technical replicates per experiment, unless otherwise noted.

For analysis of biofilm growth kinetics, beads were incubated for up to 28 days as described above. At days 3, 7, 10, 14, 21 and 28, three beads were removed and used for quantification of CFU/bead as described below.

To test the reproducibility of bacterial counts in biofilm attached to a bead, five experiments were conducted. Each strain was grown for 21 days on glass beads, after which the bacteria were detached and enumerated as described below.

For determination of CFU/bead, the beads were taken from the 24-well plate, carefully dipped twice into sterile water to wash off loosely attached bacteria, and transferred to a tube containing 2 mL of sterile water. The samples were sonicated and homogenized in an ultrasonic bath (BactoSonic®, Bandelin, Berlin, Germany) at 40 kHz and 200 W_eff_ for 20 min to detach the biofilm from the bead and disperse the bacteria. The samples were plated in serial dilution by 5 µL spot-plating on Middlebrook 7H10 agar plates supplemented with 10% OADC (BD, Heidelberg, Germany; MB-OADC). Plates were incubated for 21—28 days at 37 °C, after which the colonies were counted.

### Disinfectant efficacy testing

Efficacy testing was performed for three disinfectants (glutaraldehyde (GA), chlorine (free active chlorine, FAC), and peracetic acid (PAA)). The concentrations tested and the respective neutralizers used in this study are listed in Table 2. The exposure time was 60 min at 20 °C in all cases. Successful disinfection was defined in accordance with EN Standard 14,348 as achieving at least 4 log_10_ reduction in mean viable CFU.

**Table 2:** List of disinfectants and corresponding neutralizers used

Disinfectant	Product	Concentrations (%)	Neutralizer
Glutaraldehyde (GA)^a^	25%, Merck, Darmstadt, Germany	0.1	1% glycine solution with 0.1% Tween 80 in water
		0.5	
		1	
		2	
		5	
Chlorine (FAC)^a^	13% free active chlorine, sodium hypochlorite, Acros Organics, Fisher Scientific, Schwerte, Germany	0.1	TSH-Thio buffer (1% Tween 80, 3% Saponin, 0.1% L-Histidin, 5% sodium thiosulfate) in PBS (0.1 M, pH 7)
		1	
		2	
		3	
		5	
Peracetic acid (PAA)^b^	Wofasteril 40%, Kesla Pharma Wolfen, Bitterfeld-Wolfen, Germany	0.01	0.5% sodium sulfite in PBS (0.1 M, pH 7)
		0.05	
		0.075	
		0.1	
		0.5	

^a^Concentration (v/v), ^b^concentration (w/v)

Prior to the experiments, the active agent (peracetic acid, glutaraldehyde, free active chlorine) of each disinfectant was titrated following standard protocols. Peracetic acid and free active chlorine were titrated directly before each experiment, while glutaraldehyde was titrated once before all experiments.

### Disinfection assays for biofilm

Disinfectant efficacy testing on *M. chimaera* biofilms was performed according to the Bead Assay [21]. Biofilms were cultured for 21 days at 37 °C on porous glass beads. This time point represents the most uniform and stable growth among all strains. After treatment with disinfectants, the surviving bacteria were quantified by counting CFU/bead as described above.

### Disinfection assays for bacteria in suspension

The bacteria for suspension testing were cultivated following the EN 12353 for mycobacteria. Briefly, the stock culture was inoculated on five MB-OADC plates and incubated at 37 °C as long as necessary for analysis. Disinfection was performed according to EN 14348. Briefly, bacteria were washed three times with 0.1% Tween 80, centrifuged, and the pellet was weighed, resuspended in 0.1% Tween 80 solution, and homogenized in an ultrasonic bath (BactoSonic®, Bandelin, Berlin, Germany) at 40 kHz for 20 min. For each concentration, 900 µL of disinfectant was added to a 2 mL tube and preheated to 20 °C for 10 min. 100 µL of suspension test solution was added to the disinfectant and incubated at 20 °C for 60 min. After incubation, 100 µL of the suspension-disinfectant mixture was transferred to a new tube containing 900 µL of cold neutralizing agent. The samples were processed in serial dilution by 5 µL spot-plating on MB-OADC plates and incubated for 28 days at 37 °C.

### Weekly PAA disinfection of *M. chimaera* ZUERICH-1 biofilm

Biofilm of *M. chimaera* ZUERICH-1 was cultivated in sterile-filtered tap water or in MB-OADC as described above for 21 days. Thereafter, the biofilm beads were treated with 0%, 0.045%, 0.1% or 0.5% PAA for 10 min at room temperature. Three beads per concentration were washed by dipping in sterile water, and after neutralization the CFU counts were determined as described above. The other beads were washed by dipping twice in sterile water, transferred to new 24-well plates containing fresh sterile-filtered tap water, and incubated again on an orbital shaker at 150 rpm at 37 °C for seven days. The procedure was repeated in a 7-day cycle over three weeks, treating each biofilm bead with the corresponding PAA concentration as in the previous week.

### Neutralization and toxicity testing

Prior to disinfectant testing, complete neutralization as well as exclusion of toxicity of the neutralizing agents had been verified with the maximal concentration of disinfectant used (Table 2). All neutralizing agents were freshly prepared before the experiment. Testing was performed as described by Konrat et al. [21]. Samples were serially diluted and 100 µL of each sample were inoculated to MB-OADC plates and incubated for 14—28 days at 37 °C.

### Calculating the Reduction factor

In order to compare the results of different strains and disinfectants, the reduction factor was calculated by comparing the CFU of treated samples with the CFU of untreated samples.

The reduction factor (efficacy of disinfectant) was calculated by the equation RF = N_0_—N (RF = reduction factor; N_0_ = log_10_ CFU/bead of untreated control; N = log_10_ CFU/bead of treated sample). The arithmetic means and standard deviations of reduction were calculated from three biological replicates.

### Scanning Electron Microscopy (SEM)

The biofilm samples were cultivated for 21 days as described above. The samples were fixed (4% paraformaldehyde, 2.5% glutaraldehyde in 50 mM HEPES, pH 7.0) for 48 h at 4 °C, dehydrated in a graded ethanol line (30, 50, 70, 90, 95, 100, 100%), dried overnight in hexamethyldisilazane, mounted on aluminum stubs, sputter coated with 16 nm gold–palladium and examined in the SEM (ZEISS 1530 Gemini, Carl Zeiss Microscopy GmbH, Germany) operating at 3 kV using the in-lens electron detector. Images were cropped and adjusted for optimal brightness and contrast (applied to the whole image) using Adobe Photoshop® (Adobe Systems, San Jose, CA, USA).

### Biomass measurement of ECM components

#### Batch Sample preparation

Biofilms were grown for 21 days as described above. To produce a batch sample, biofilm material from 24 porous glass beads was pooled in a falcon tube containing 50 mL sterile water. For the suspension batch sample, five agar plates were washed with 10 mL sterile water each and resuspended bacteria were transferred to a 50 mL falcon tube. For enumerating the bacteria, samples were sonicated for 20 min at 40 Hz in an ultrasonic bath to individualize the bacteria and CFU counts were determined as described above.

These experiments were performed five times, thus representing five biological replicates per strain.

#### Quantification of protein

A quantified standard of mycobacterial proteins was produced as described by Lewin et. al [22]. The amount of protein was determined using the BCA Protein Assay (Pierce) according to the manufacturer’s instructions. 50 µL of each biofilm or suspension batch sample, respectively, was transferred to a separate well of a clear bottom 96—black well plate (Greiner Bio-One International). An additional 50 µL of the serial dilutions of the mycobacterial protein standard were also transferred to the 96—well plate. Each sample and standard were processed in three technical replicates.

50 µL of staining solution (DMSO with 0.2% SYPRO™ Orange protein gel stain (Thermo Fisher Scientific, Waltham, MA USA) was added to each well and mixed carefully. The plate was incubated at room temperature for 30 min in the dark and the fluorescence (470Ex/570Em) was subsequently measured in a TECAN microplate reader (TECAN Trading). The protein mass per well was calculated using a standard curve generated from the mycobacterial protein standard and related to the CFU/mL of the sample.

#### Quantification of lipid

10 mL of each sample were transferred to a 50 mL falcon tube and centrifuged at 8000 × g and 4 °C for 10 min. The supernatant was discarded and the pellet resuspended in the 20-fold volume of 2:1 chloroform/methanol. The tubes were sonicated for 20 min at 40 Hz and incubated at room temperature overnight. The samples were centrifuged again and the supernatant transferred to a pre-weighed 50 mL tube and evaporated completely. The falcon tube was weighed again and the mass of lipid per bacteria was calculated using the CFU/mL. Each sample was processed in three technical replicates.

#### Quantification of DNA

Preparation of DNA standard for qPCR was performed according to the method described by van Soolingen et al. [23] with modifications. Briefly, one milliliter of culture was centrifuged, and the pellet resuspended in 400 μL TE buffer (0.01 M Tris–HCL, 0.001 M EDTA, pH 8.0). After heating at 80 °C for 30 min, 5 μL lysozyme (150 mg/mL) was added and incubated overnight at 37 °C. Subsequently, 70 μL 10% SDS and 2 μL proteinase K (50 mg/mL) were added, followed by incubation at 65 °C for 2 h. Then, 100 μL 5 mM NaCl and 100 μL CTAB (Cetyltrimethylammoniumbromid) buffer (10% CTAB in 0.7 M NaCl) were added and incubated for 10 min at 65 °C. DNA was extracted with chloroform/isoamylalcohol and phenol/chloroform/isoamylalcohol, precipitated with isopropanol, washed with 70% ethanol, dried, and resuspended in TE buffer.

The preparation of samples for qPCR followed the description of Lewin et. al [24]. Briefly, 100 µL from each biofilm batch or suspension batch samples were taken and DNA was made available for PCR by heating the bacteria for 30 min at 96 °C. Primers and probe were designed on the *rpoB* gene using the genome sequence of *M. chimaera* ZUERICH-1 [11] (Genbank accession CP015272.1). The primers and the probe were designed in this study using the software Geneious Prime ® (2020.2.3, Biomatters, Ltd., New Zealand): forward: 5´-TGGACCAACGAGCAGATCAC-3´ (GC 55%, melting temperature 61.5 °C); reverse: 5´-TGTTGTCCTTCTCCAGCGTC-3´ (GC 55%, melting temperature 61.3 °C); probe: 5'-FAM-GGCTTCTCCGAGATCATGATGT-TAMRA-3' (GC 50%, melting temperature 61.07 °C); product size 73 bp. A quantitative TaqMan-PCR was performed as described before [25] using an Agilent AriaMx Real-time PCR system (Agilent, Santa Clara, USA) with the following thermal profile: 10 min 95 °C, followed by 40 cycles of 30 s 95 °C, 30 s 60 °C, 1 min 72 °C. Using a standard curve of quantified isolated DNA samples, the resulting Cq/∆Cq was analyzed to quantify the amount of DNA (in pg/mL) for each sample. The amount of DNA per bacterium was calculated using the CFU/mL.

### Statistical analysis

Statistical analysis was performed using R (3.6.1 GUI 1.70 El Capitan build) and RStudio© (1.2.5019; RStudio, Inc). Statistical significance was verified using Wilcoxon-Rank-Sum test (Mann–Whitney-U-test) under the assumptions of randomly sampled and unpaired data. Level of significance is indicated by *p*-value (* = α < 0.05; ** = α < 0.01; *** = α < 0.001).

## Results

### Biofilm growth

**Fig. 1:**
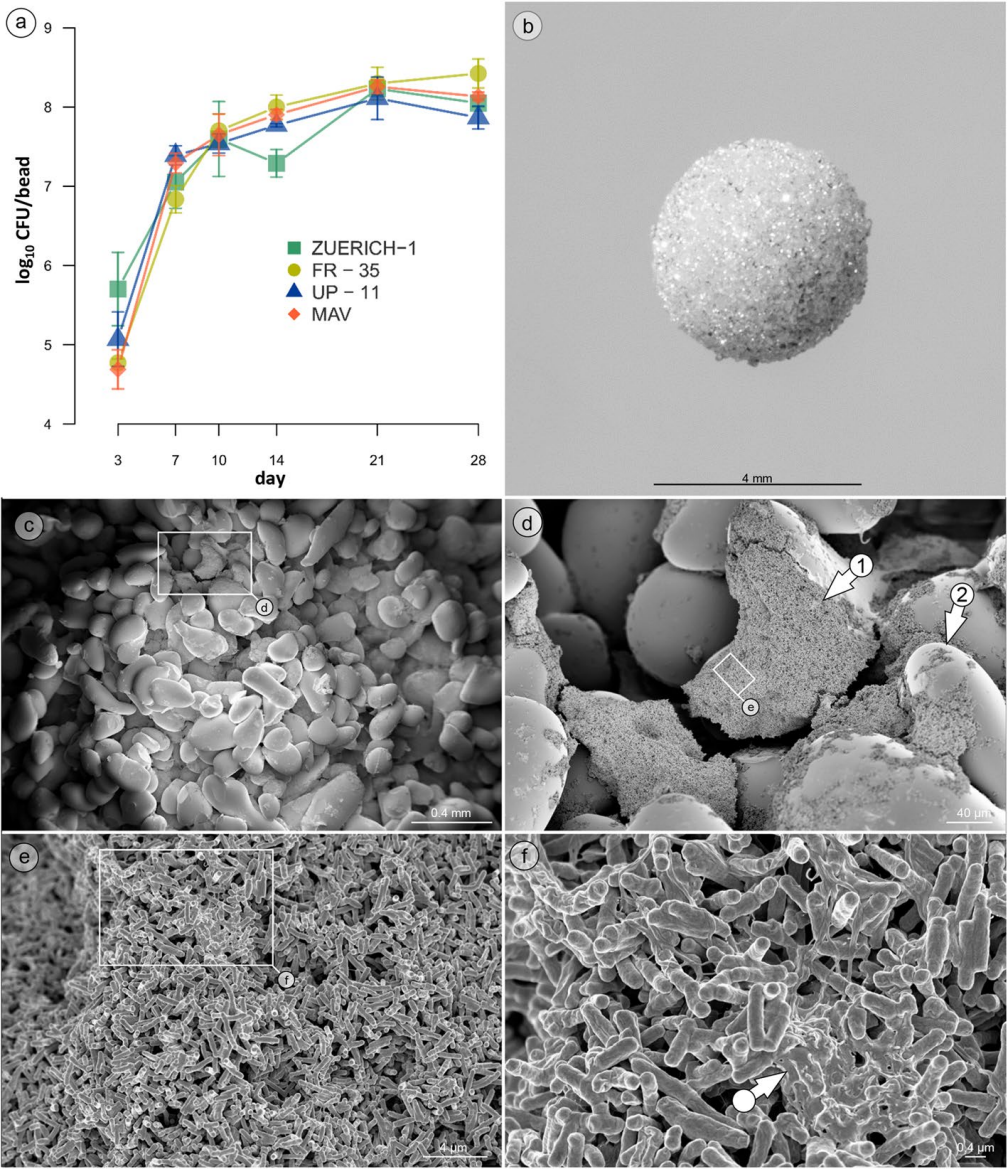
Biofilm growth on porous glass beads. **a** Biofilm growth curves of *M. chimaera* (ZUERICH-1, FR-35, UP-11) and *M. avium* subsp. *avium* (MAV) strain ATCC 15769. **b** Macrophotography of a porous glass bead. **c** SEM image showing the overall distribution of the ZUERICH-1 biofilm on the porous glass bead. **d** The biofilm is mainly composed of macro-colonies (arrow 1), which have grown on and between the glass sinter particles (arrow 2). **e** In these macro-colonies, *M. chimaera* is arranged in densely packed multi layers of bacteria, which are **f**) partially embedded in an extracellular slime matrix (arrow)

All tested strains were able to form biofilm on the porous glass beads (Table 1). The growth curves showed a consistent growth pattern among all strains (Fig. 1a). From day 3 to 10, the biofilms showed exponential growth that flattened after day 14 to a pattern of almost stationary growth. At day 21, the strains had a nearly identical number of bacteria per bead (approx. 8 log_10_) with small differences between samples.

The analysis of the CFU/bead of five biological replicates per strain after 21 days proved that all four strains produced a reproducible amount of biofilm (Supplementary Fig. 1). The means of all five biological replicates did not differ more than 0.5 log_10_ from the overall mean, thereby fulfilling the prerequisites for disinfectant testing (according to EN 14348 for basic limits).

### Tolerance of biofilms to disinfectants

**Fig. 2:**
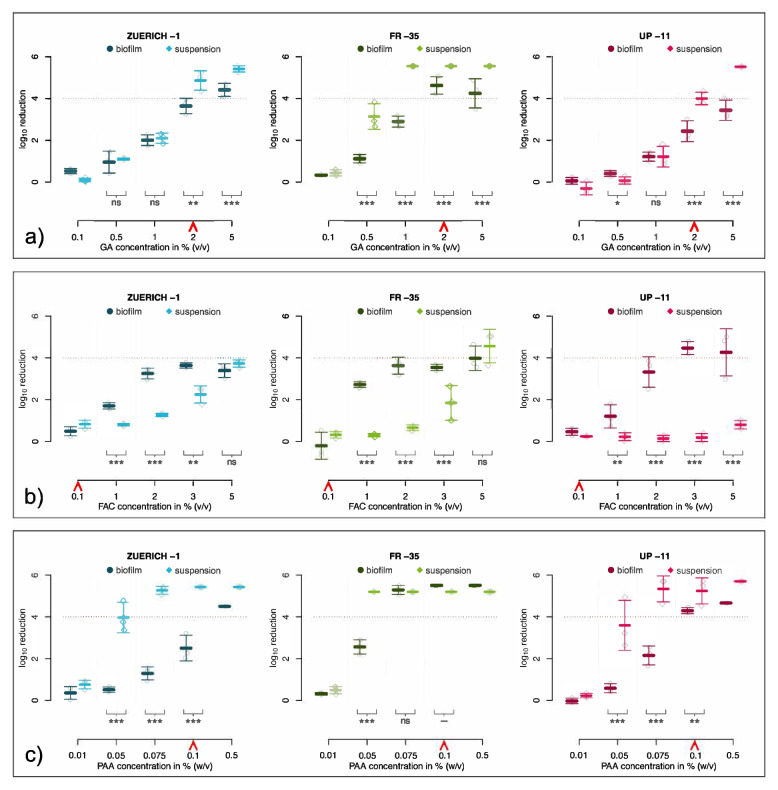
Disinfectant efficacy testing of *M. chimaera*. Disinfectant testing of biofilm and suspension of three different *M. chimaera* strains with glutaraldehyde (**a**), free active chlorine (**b**) and peracetic acid (**c**). In comparison to the suspension, the biofilms of all strains show enhanced tolerance against GA and PAA. With regards to FAC, the suspension of all three strains was more tolerant than the corresponding biofilm in concentrations ≧ 1%. Lines indicate the calculated mean reduction and corresponding standard deviation of three biological replicates depending on the untreated control. Dotted line displays reduction threshold of 4 log_10_. Red arrows indicate standard disinfectant concentrations. Statistical significance was calculated using Wilcoxon-Rank-Sum test (*p* < 0.05 = *; *p* < 0.01 = **; *p* < 0.001 = ***; ns = not significant)

Disinfectant efficacy testing with GA showed that in higher concentrations (≥ 2% GA), biofilm formation leads to a significantly higher tolerance in all strains. At low and intermediate concentrations of GA, the tolerance of biofilm and bacteria in suspension was mostly similar (Fig. 2a).

Effective disinfection was not achievable for biofilms or suspensions of the ZURICH-1 strain at any FAC concentration tested. Biofilm and suspension of *M. chimaera* FR-35 were inactivated at 5% FAC. Biofilm of *M. chimaera* UP-11 was inactivated at 3% FAC, while the suspensions, even at the highest concentration tested, were not reduced by more than 1 log_10_. The suspensions of all three *M. chimaera* isolates showed a significantly higher tolerance at concentrations from 1–3% FAC in comparison to their biofilms (Fig. 2b).

For PAA, the biofilm of *M. chimaera* ZUERICH-1 was effectively inactivated only at the highest concentration (0.5%), while the suspension was far more sensitive, showing effective reduction in CFU at 0.075%. The biofilms of the other *M. chimaera* strains tested also showed an enhanced tolerance to PAA when compared to the suspensions, although no biofilm was as tolerant to PAA as *M. chimaera* ZUERICH-1 (Fig. 2c).

The results of the disinfectant testing for *M. avium* subsp. *avium* ATCC 15769, which was used as a reference strain, were similar to those for the *M. chimaera* strains. *M. avium* biofilms also displayed a remarkable tolerance to disinfection by GA and PAA, but not to FAC (Suppl. Figure 2). We compared the results of suspension testing for *M. avium* subsp. *avium* ATCC 15769 to internal controls to verify the reliability of the results (data not shown).

Standard concentrations of the disinfectants that are recommended for the decontamination of HCU or surgical instruments, i.e. 2% GA, 0.1% PAA and 0.1% FAC, did not sufficiently eradicate the biofilm of *M. chimaera* ZUERICH-1 (Table 3). In contrast, 0.1% peracetic acid effectively inactivated biofilms of the other two *M. chimaera* strains and the suspensions of all three strains tested. Chlorine failed in its recommended concentration of 0.1% to effectively inactivate any of the strains tested in biofilm or in suspension.

**Table 3:** Efficacy of standard disinfectant concentrations used in HCU on *M. chimaera* strains. Successful reduction (≥ 4 log_10_) is indicated by “ + ”, insufficient reduction (< 4 log_10_) is indicated by “- “. The exposure time was in all cases 60 min at 20 °C

Strain		2% GA*	0.1% FAC	0.1% PAA
*M. chimaera ZUERICH-1*	Biofilm	-	-	-
	Suspension	+	-	+
*M. chimaera FR-35*	Biofilm	+	-	+
	Suspension	+	-	+
*M. chimaera UP-11*	Biofilm	-	-	+
	Suspension	+	-	+

^*^Concentration of GA is derived from recommendations for endoscope standard disinfection

### Biofilm architecture

Electron microscopic imaging showed that all tested strains formed an adhesive biofilm on the outer surfaces as well as in the pores of the porous glass beads (Fig. 1c - d; Supplementary Fig. 3). Upscaled images of *M. chimaera* ZUERICH-1 showed the formation of aggregation structure embedded in a slime matrix in and outside of the pores (Fig. 1c - f).

### ECM composition

**Fig. 3:**
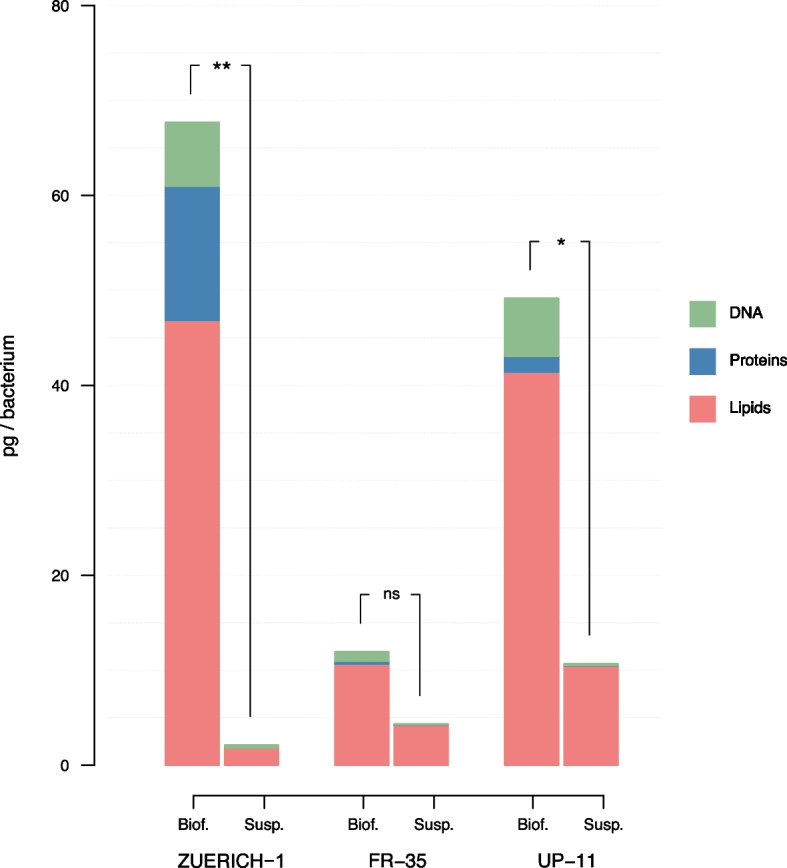
Comparison of biomass compositions. The analysis of the amount of the biomass components proteins, lipids and DNA in biofilm and suspension samples of the *M. chimaera* strains. ZUERICH-1 produced more overall biomass than the other strains and its biofilm contained over five times more proteins than the other biofilms. The amount of DNA in pg/bacterium did not differ much among ZUERICH-1 and UP-11. FR-35 showed almost no additional DNA in biofilm compared to suspension. ZUERICH-1 and UP-11 showed an enhanced mass of lipids in their biofilms in comparison to FR-35. The bars of each component represent the mean of five biological replicates. The total mass of ECM components was compared between biofilm and suspension of each strain by Wilcoxon-Rank-sum test (ns = not significant; * = *p* < 0.05; ** = *p* > 0.01)

The biofilms of all tested strains showed generally higher amounts of each ECM component tested (lipids, protein, DNA) when compared to bacteria in suspension (Fig. 3). *M. chimaera* ZUERICH-1 biofilm showed the highest total amount of ECM in comparison to the other strains. The difference between mass of extracellular components in biofilm vs. suspension was significantly greater in *M. chimaera* ZUERICH-1 than in the other two tested strains.

In all *M. chimaera* biofilm samples, the ECM primarily consisted of lipids (> 60% total mass of ECM).

*M. chimaera* ZUERICH-1 biofilm produced approx. 15 pg protein per bacterium, which is over fivefold the amount produced by the other strains; and in relation to the total mass of ECM, ZUERICH-1 possessed the highest proportion of proteins (Fig. 3).

The amount of DNA in the tested strains was higher in the biofilm than in the suspension, with the exception of *M. chimaera* FR-35, which showed almost no additional production of DNA in biofilm.

### Weekly disinfection of *M. chimaera* ZUERICH-1 biofilm

**Fig. 4:**
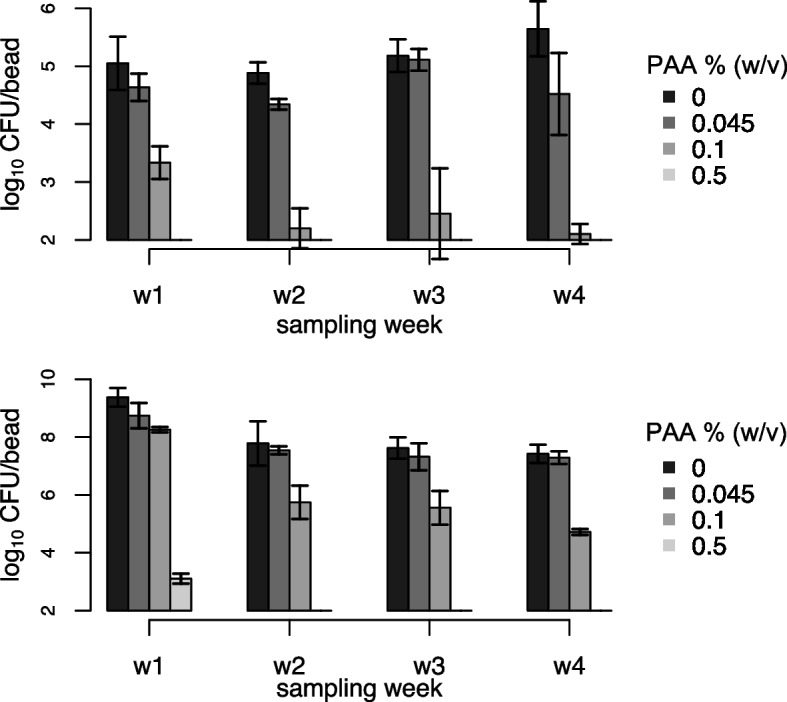
Weekly disinfection. Biofilms of *M. chimaera* ZUERICH-1 were cultured for 21 days in sterile-filtered tap-water (**a**) or in MB-OADC (**b**) and disinfected afterwards in weekly cycles with different concentrations of PAA for 10 min. Bars represent the mean CFU/bead with standard deviation of three biological replicates

Biofilms were treated once a week for 10 min with different concentrations of PAA in order to assess the long-term impact of the recommendations for repeated routine disinfection of certain types of HCU (LivaNova; Operating Instructions Feb. 2020). We found that neither 0.045% nor 0.1% PAA could achieve full eradication or effective reduction (≥ 4 log_10_) of biofilms of ZUERICH-1 over a time period of 4 weeks (Fig. 4). In detail, disinfection using 0.045% PAA showed little reduction (0.5–1 log_10_) in CFU when compared to the control. In the first week, disinfection with 0.1% PAA achieved a reduction in the biofilm of 1.5 log_10_ CFU/mL. Interestingly, the number of surviving bacteria was lower in the second week, but remained constant in the following weeks despite repeated disinfection with 0.1% PAA.

Only disinfection with the highest concentration tested (0.5% PAA) was found to be effective in reducing the biofilm CFU below the detection limit in the first week and thereafter.

## Discussion

The outbreak of *M. chimaera* infections related to HCU emphasized the importance of nosocomial mycobacterial infections and the need to provide guidance for their diagnosis and prevention, as well as a call for future research [11]. Our study investigated the disinfecting efficacy of different compounds towards *M. chimaera* biofilms.

### Recommended disinfectants of HCU are not sufficient to eradicate *M. chimaera* in biofilm

At the beginning of the outbreak in 2013, manufacturers recommended a concentration of 0.045% peracetic acid or 0.02% sodium hypochlorite for the disinfection of water circuits by floating the tanks and tubes of HCU for 10 min [12]. Revised disinfection guidelines recommended regular disinfection with 0.1% peracetic acid or 0.1% sodium hypochlorite for 10 min [26].

In our experiments, biofilm of *M. chimaera* ZUERICH-1 displayed tolerance to concentrations of up to 0.5% PAA during 60 min exposure, which by far exceeds the recommended disinfection procedures. This was also true for FAC, with which neither the biofilm nor suspension of ZUERICH-1 was reduced sufficiently by a concentration of up to 5% FAC for a duration of 60 min.

Interestingly, the ZUERICH-1 strain displayed a remarkably increased tolerance to PAA when compared to the other *M. chimaera* strains tested. This increase might have resulted from adaptation to a repeated or continuous sublethal concentration of PAA and FAC in the HCU; this has already been shown in previous studies with biofilms and antimicrobials [18]. In *M. avium* subsp. *hominissuis* (MAH), promising metabolic pathways have recently been identified that could be targeted to prevent a mycobacterial tolerance mechanism [27]. Whether this mechanism may also contribute to the increased tolerance to disinfectants in biofilms of *M. chimaera* is subject to future studies.

In order to compare our results more directly to the conditions found in HCU, we tested the tolerance of *M. chimaera* ZUERICH-1 biofilm grown in sterile-filtered tap water to a weekly disinfection with PAA. We found that even the updated recommendations of using 0.1% PAA for 10 min did not fully reduce or eradicate the bacteria despite their cultivation in sub-optimal conditions.

These results suggest that the disinfection recommendations of medical devices containing water circuits need further revision. This, however, presents a potential problem, as increasing the concentration of disinfectant and/or the contact time needed for complete eradication of *M. chimaera* could presumably harm the machines’ internal components and lead to impaired function and integrity of the HCU. Alternative strategies, such as a vacuum seal to close the ventilation system or the use of a glycol-based heat transfer fluid instead of water, may be more successful in preventing bioaerosols transmission. Another interesting aspect would be to focus on the manufacturing site, where the primary contamination presumably happens. Recommendations for the manufacturer to evaluate NTM species and strains as part of the microbial risk assessment during device development and prior to regulatory submission would help to reduce the risk of contamination of devices and thus of future outbreaks.

The contamination of medical devices with NTM is not exclusive to HCU. Trudzinski et. al. [28] showed that 50% of ECMOs (extracorporeal membrane oxygenation machines) in a single hospital were contaminated with *M. chimaera*. The contamination was often linked to the colonization of water tanks or water supplies by these ubiquitous bacteria [29–31].

Interestingly, we found that in concentrations of free active chlorine ≥ 1%, all suspensions showed a higher tolerance than the corresponding biofilm. This phenomenon might be explained by the availability of oxygen in the cultivation process. Falkinham [32] showed that oxygen accessibility during the cultivation of *M. avium* has a significant influence on the susceptibility towards chlorine. Bacteria exposed to oxygen-reduced growth conditions were less tolerant than those grown in ambient air. We assume that different culture conditions of suspension and biofilm samples resulted in differences in oxygen availability. While bacteria grown on agar plates are largely well supplied with oxygen, bacteria in biofilm often face hypoxia, especially bacteria in the inner parts of larger aggregates. The expression of genes linked to hypoxia is commonly observed in biofilms [33–35].

The present results are based on a small number of tested strains. It was not expected that the suspensions exhibit an enhanced tolerance towards FACS compared to biofilm. It could not be finally clarified, whether the genetic profile of the strains, the hypoxic conditions in parts of the biofilms or the composition of the ECM components are primarily responsible for the enhanced tolerance of the suspension towards FAC. Further evaluation has to be focused on these influencing factors and a larger number of strains should be tested to exclude potential coincidental results.

Other limitations of our study are that the experiments were conducted at room temperature (20 °C) and with biofilms of a single species. Therefore, the results cannot be extrapolated to other temperature conditions or to biofilms of multiple species. Further studies are needed to better understand the impact of these conditions on disinfection efficacy.

### *M. chimaera* ZUERICH-1 produces high amounts of ECM

In comparison to suspension, the ZUERICH-1 biofilm showed a strong increase in biomass of ECM. Interestingly, this difference was larger in ZUERICH-1 than in the other two strains. The amount of biomass in the biofilm samples of ZUERICH-1 and UP-11, a clinical isolate, was not significantly different. However, both strains produced significantly more ECM components per bacterium in biofilm than FR-35, which could explain the lower tolerance of this strain to disinfectants. The amount of biofilm produced by ZUERICH-1 and UP-11 could be an adaptation triggered during infection to provide better protection against the patient's immune response. Further research is needed to determine whether this is true or purely coincidental, as our study is limited to only three strains, which may not be representative for all *M. chimaera* strains. Another important factor are the culturing conditions which probably influence the composition and the amount of the ECM as we have shown before for *M. abscessus* [20]. The effect of the culturing conditions should be focused in further disinfection studies of mycobacterial biofilms.

Another distinctive feature of ZUERICH-1 biofilm was the increased amount of proteins, which was over fivefold more than the amounts present in the biofilms of the other isolates. In *Salmonella* biofilms, it has been shown that exposure to a sub-lethal concentration of benzalkonium chloride led not only to an increase in tolerance, but also to an adaptation of the biofilm matrix [36]. The increased production of proteins in ZUERICH-1 could therefore be an adaptation to a longstanding exposure to sub-lethal concentrations of disinfectants in HCU. The high protein content in the ECM could explain the enhanced tolerance of the biofilm of *M. chimaera* ZUERICH-1 towards PAA. It has been shown that the tolerance to disinfectants depends on whether the composition of the matrix is dominated by proteins or polysaccharides, as these tend to be more reactive to disinfectants and thus lead to higher consumption [37–39]. Packing density and the metabolic state of the bacteria are also important factors that influence tolerance to disinfectants. Steed et al. [40] investigated the effect of disinfectants on intact and disrupted biofilms in comparison to planktonic *M. avium* and *M. intracellulare* and found that structurally intact biofilms were more tolerant.

It is well known that the biofilm´s structure plays an important role in conferring tolerance. Our microscopy results suggest that the different location of the bacteria on more or less exposed areas of the porous glass beads, and thereby the strength of the shear forces acting on them, influences the density of the matrix and the overall structure of the biofilm aggregates. Bacteria attached to outer parts of the glass particles displayed a very dense and slimy packing in ECM, while bacteria in the inner bead areas showed less matrix production but larger, multilayered structures. Interestingly, *M. chimaera* ZUERICH-1 was the only strain which displayed net-like structures that covered parts of the biofilm-associated bacteria. The chemical composition of these structures and their potential role in tolerance to disinfectants need further investigation, as well as the structural and compositional changes that presumably occur when cultivation is performed with multi-species biofilms.

In conclusion, our data show that *M. chimaera* ZUERICH-1 biofilm cannot be sufficiently inactivated by the disinfectants and conditions that are currently recommended for the disinfection of HCU. The *M. chimaera* ZUERICH-1 outbreak strain displays distinct characteristics such as enhanced production of proteins in biofilm that might explain its increased tolerance to disinfectants. The FDA continues to evaluate the risks of NTM infection through HCU and developed additional measures to reduce their contamination [41]. The recommendations for the disinfection of water-carrying appliances should consider the risk of biofilm formation and provide effective protection against biofilm-associated microorganisms.

## Supplementary Information

Supplementary Material 1.

## Data Availability

The datasets used and/or analyzed during the current study are available from the corresponding author on reasonable request.

## Abbreviations

CFU: Colony forming units
ECM: Extracellular matrix
FAC: Free active chlorine
GA: Glutaraldehyde
HCU: Heater cooler Unit
MAC: *Mycobacterium avium* Complex
NTM: Nontuberculous mycobacteria
PAA: Peracetic acid
PGB: Porous glass bead
v/v: Volume/volume
w/v: Weight/volume
