# Supplementary material for: Biofilm formation by the global outbreak strain of Mycobacterium chimaera results in significantly reduced efficacy of standard disinfectants
Source: BMC Microbiol. 2025 Nov 13;25:738. doi: 10.1186/s12866-025-04439-w (PMC12613882; doi:10.1186/s12866-025-04439-w)
Supplement: Supplementary file 1 — Supplementary Material 1. [file 12866_2025_4439_MOESM1_ESM.pdf]

Supplementary

Biofilm formation of Mycobacterium chimaera results in reduced efficacy of standard disinfectants.
Oschmann et al.

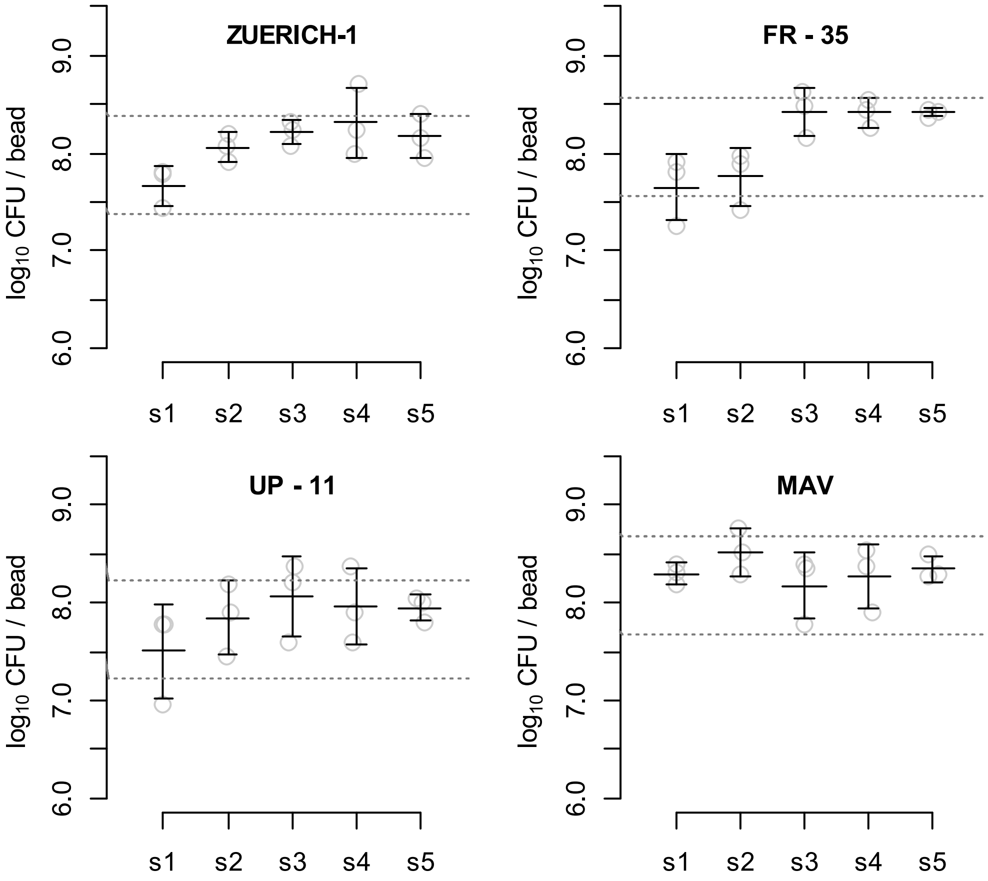

Figure 1 - Reproducibility

Reproducibility of biofilm growth on porous glass beads from five biological samples (s1 - s5), each in three technical replicates. Bars represent the mean and standard deviation; light grey circles represent technical replicates for each sample. Grey dotted lines indicate a maximal tolerated variance between means of samples (+/- 0.5 log_{10} from the overall mean). All sample means are within the tolerated variance calculated for each strain.

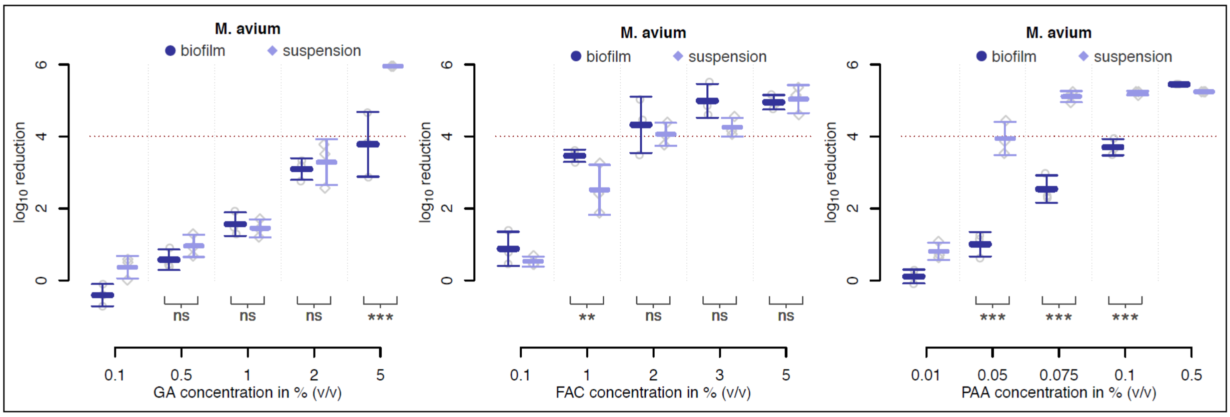

Figure 2 - *M. avium* disinfectant testing

The strain *M. avium* ATCC 15769 was tested as a reference strain to double check results from *M. chimaera* disinfection testing.

Tolerances of biofilm and suspension were very similar when disinfected with glutaraldehyde. When disinfected with FAC the suspension was slightly more tolerant than the biofilm. Against PAA the biofilm of *M. avium* ATCC 15769 was significantly more tolerant than the suspension. Lines indicate the calculated mean reduction and corresponding standard deviation of three biological replicates depending on the untreated positive control. Dotted line displays reduction threshold of 4 log_{10} steps. Red arrows indicate standard disinfectant concentrations. Statistical significance was calculated using Wilcoxon-Rank-Sum test ($p < 0.05$ = *; $p < 0.01$ = **; $p < 0.001$ = ***; ns = not significant).

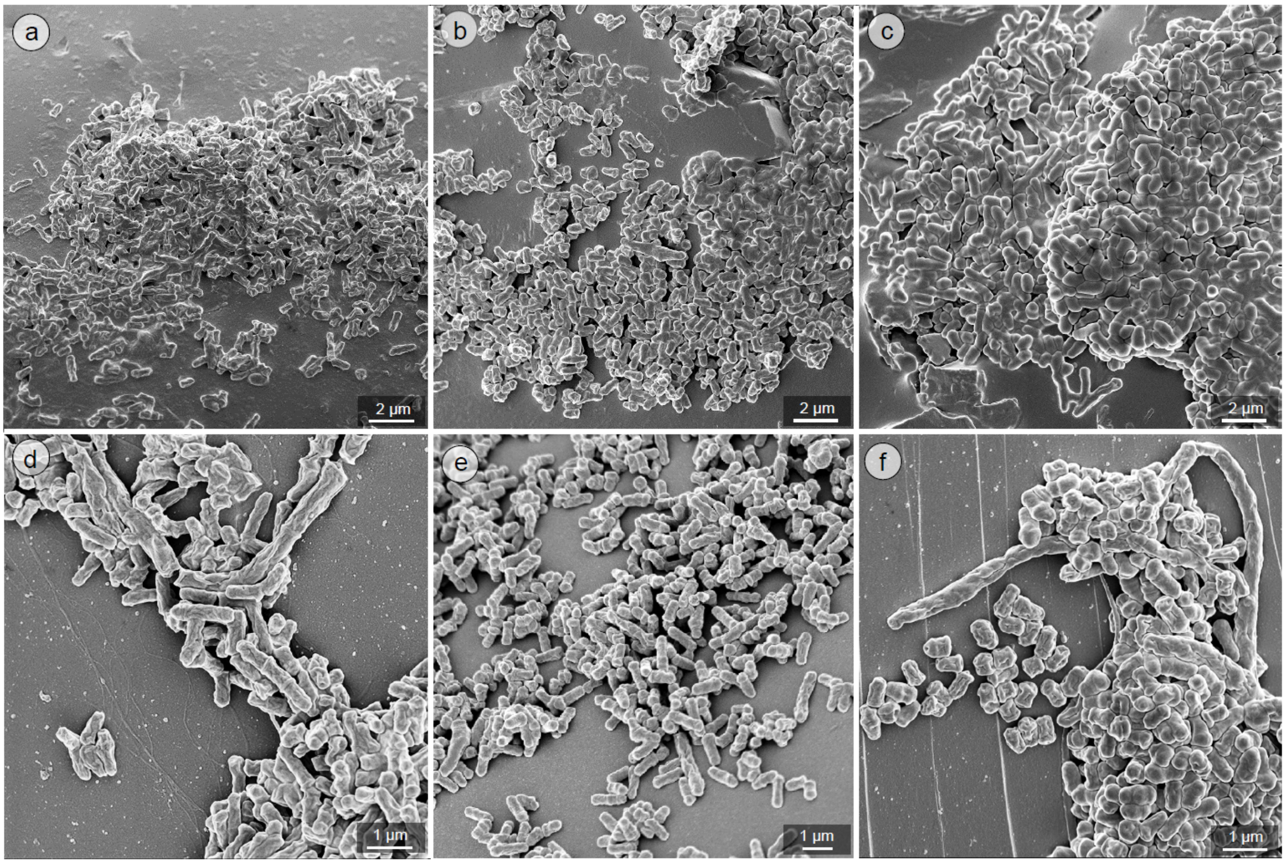

Figure 3 – Biofilm of *M. chimaera* ZUERICH-1, FR-35 and UP-11

While some areas of the different *M. chimera* biofilms are heavily embedded in their own slime matrix – a) Zuerich-1, b) FR-35, c) UP-11 - other regions reveal earlier stages of biofilm formation with less visible traces of such an extra cellular matrix around the mycobacteria (d) ZUERICH-1, e) FR-35, f) UP-11).
